# Demethylation of HIN-1 reverses paclitaxel-resistance of ovarian clear cell carcinoma through the AKT-mTOR signaling pathway

**DOI:** 10.1186/s12885-015-1744-5

**Published:** 2015-10-24

**Authors:** Chih-Ming Ho, Chi-Jung Huang, Shih-Hung Huang, Shwu-Fen Chang, Wen-Fang Cheng

**Affiliations:** 1 Gynecologic Cancer Center, Department of Obstetrics and Gynecology, Cathay General Hospital, Taipei, Taiwan; 2 School of Medicine, Fu Jen Catholic University, Hsinchuang, New Taipei City, Taiwan; 3 School of Medicine, Taipei Medical University, Taipei, Taiwan; 4 Department of Medical Research, Cathay General Hospital, Sijhih, New Taipei, Taiwan; 5 Department of Pathology, Cathay General Hospital, Taipei, Taiwan; 6 Graduate Institute of Medical Sciences, School of Medicine, Taipei Medical University, Taipei, Taiwan; 7 Department of Obstetrics and Gynecology, National Taiwan, University Hospital, Taipei, Taiwan; 8 Graduate Institute of Oncology, National Taiwan, University Hospital, Taipei, Taiwan; 9 Graduate Institute of Clinical Medicine, College of Medicine, National Taiwan, University, Taipei, Taiwan; 10 Department of Biochemistry, National Defense Medical Center, Taipei, Taiwan

**Keywords:** Ovarian clear cell carcinoma, 5-aza-2-deoxycytidine, HIN-1, AKT/mTOR, Hypoxia-inducing factor

## Abstract

**Background:**

Methylation of HIN-1 is associated with poor outcomes in patients with ovarian clear cell carcinoma (OCCC), which is regarded to be an aggressive, chemo-resistant histological subtype. This study aimed to evaluate whether 5-aza-2-deoxycytidine (5-aza-2-dC) can reverse methylation of the HIN-1 gene to restore chemo-sensitivity of OCCC and the possible mechanism.

**Methods:**

*In vitro* flow cytometric analysis and evaluation of caspase-3/7 activity of paclitaxel-sensitive and resistant OCCC cell lines were performed. Methylation status and expression changes of HIN-1 in the OCCC cell lines treated with 5-aza-2-dC were evaluated, and immunohistochemical staining of HIN-1 in OCCC tissues was performed. *In vivo* tumor growth with or without 5-aza-2-dC treatment was analyzed, and Western blotting of AKT-mTOR signaling-related molecules was performed.

**Results:**

G2-M phase arrest was absent in paclitaxel-resistant OCCC cells after treatment with the cytotoxic drug. The caspase activities of the chemo-resistant OCCC cells were lower than those of the chemo-sensitive OCCC cells when treated with paclitaxel. Methylation of HIN-1 was noted in paclitaxel-resistant OCCC cell lines and cancerous tissues. 5-aza-2-dC reversed the methylation of HIN-1, re-activated the expression of HIN-1, and then suppressed the i*n vivo* tumor growth of paclitaxel-resistant OCCC cells. Immunoblotting revealed that phospho-AKT473 and phospho-mTOR were significantly increased in HIN-1-methylated paclitaxel-resistant OCCC cell lines. However, the expressions of phospho-AKT at Ser473 and Thr308 and phospho-mTOR decreased in the OCCC cells with a high expression of HIN-1.

**Conclusions:**

Demethylating agents can restore the HIN-1 expression in paclitaxel-resistant OCCC cells through the HIN-1-AKT-mTOR signaling pathway to inhibit tumor growth.

## Background

Ovarian carcinoma is the fourth most common cause of cancer death among women in the United States [1]. Cytoreductive surgery followed by platinum-based chemotherapy is the standard initial treatment and has improved survival in patients with ovarian cancer [2]. Recently, ovarian clear cell carcinoma (OCCC) has become the second most common subtype in North America, and the second leading cause of death from ovarian cancer [3]. The overall incidence of OCCC has been reported to be higher in Taiwan and Japan [4–6].

The combination of paclitaxel and platinum, recognized as the gold standard regimen for ovarian cancer [7], is used to treat patients with all subtypes of ovarian neoplasms including OCCC. Compared to ovarian serous carcinoma, OCCC is relatively resistant to platinum or taxane-based chemotherapy, and this chemo-resistance is associated with a lower response rate to chemotherapy and a poor prognosis [5, 6, 8, 9]. For second-line or salvage treatment, the response rate for recurrent or refractory OCCC is far lower than that for other histological tumors, and even in patients with platinum-sensitive OCCC the response rate is lower than 10 % [10]. Therefore, in order to improve the survival of patients with OCCC, the development of novel treatment strategies for both first-line and salvage treatment for recurrent disease is urgently needed. To achieve this goal, the identification of targets associated with chemo-resistance and elucidation of the molecular mechanisms of this process are urgently required.

Candidate DNA methylation drivers of acquired cisplatin resistance in ovarian cancer identified by methylome and expression profiling has been reported recently [11]. Data on potential key drivers of chemo-resistance in OCCC that are silenced by DNA methylation are limited, and further evaluation as to their potential as therapeutic targets for drug resistance is needed. Preclinical and clinical studies strongly support the use of combination regimens, and have shown that the hypomethylating agents azacitadine and decitabine can restore platinum sensitivity in chemo-resistant ovarian cancer cell lines, xenografts, and patients with ovarian cancer [12–15]. However, the proof of concept of the therapeutic effect in parental or resistant OCCC in vitro and in vivo has not yet been established.

We hypothesized that there may be a subset of epigenetic changes causally associated with the acquisition of chemo-resistance in OCCC. In order to identify the epigenetically altered genes driving paclitaxel resistance in OCCC, we analyzed acquired DNA methylation changes in a human paclitaxel-resistant OCCC cell line, and expression changes associated with acquired resistance or following resensitization with demethylating agents. Although target therapies are currently used in many cancers, the molecular pathogenesis of chemo-resistance in OCCC is still unclear. We recently reported that methylation of HIN-1 promoter is a novel epigenetic biomarker associated with poor outcomes in patients with OCCC, and that the ectopic expression of the HIN-1 gene increases paclitaxel sensitivity partly through the Akt pathway [16]. Therefore, the aim of this study was to examine whether 5-aza-2-dC could reverse methylation of the HIN-1 gene and regulate the AKT/mTOR signaling pathway, and then restore the chemo-response to paclitaxel in OCCC.

## Methods

### Cell lines and cultures

ES2 and TOV21G cell lines were obtained from the American Type Culture Collection. All cells were maintained in a humidified atmosphere containing 5 % CO_2_ at 37 °C. ES-2 cells were grown in McCoy’s 5A medium with 10 % FBS, and TOV21G maintained in MCDB 105/medium 199 supplemented with 10 % heat-inactivated fetal bovine serum.

### Establishment of chemo-resistant tumor cell lines

Paclitaxel-resistant ES-2 and TOV21G tumor cell lines were developed by continuous exposure to paclitaxel. Briefly, ES-2 and TOV21G cells were exposed to increasing concentrations of paclitaxel, with an initial concentration of 0.001 μM. When the tumor cells regained exponential growth after paclitaxel treatment, the concentration of paclitaxel was doubled until the concentration reached 0.2 μM. The resulting paclitaxel-resistant tumor cell lines were named ES2TR160 and TOV21GTR200. The ES2TR160 and TOV21GTR200 cells were passaged weekly and treated monthly with respective concentrations of paclitaxel to maintain their paclitaxel chemo-resistance.

### Generation of HIN-1 over-expressing ES2 and ES2TR160 cell lines

ES2 and ES2TR160 tumor cells were transfected with the HIN-1 gene to generate HIN-1 over-expressing ES2 and ES2TR160 transfectants as described previously [16].

### Reagents and antibodies

ECL Western blotting detection reagents were purchased from Perkin Elmer (Boston, MA). Antibodies recognizing HIN-1, mTOR, phospho-mTOR (Ser2448), AKT, phospho-AKT (Ser473) (Thr308), and GAPDH were purchased from Cell Signaling Technology (Beverly, MA). A Cell Titer 96-well proliferation assay kit was obtained from Promega (Madison, WI), and paclitaxel was obtained from Genetaxyl Cream Less Company.

### MTT assays for cytotoxicity and proliferation assays

The sensitivities of various tumor cell lines to paclitaxel were first assessed by MTT assay. Briefly, cells (4000 cells/well) in a 96-well plate were exposed to paclitaxel at the indicated concentrations for 72 h at 37 °C. The cells exposed only to the culture medium served as controls. MTT at a final concentration of 0.5 mg/ml was added to the cells and incubated at 37 °C for 3 h. At the end of incubation, the cultured medium was removed and 200 μl of DMSO (Sigma) was added to dissolve the blue formazan crystals, and the optical density was measured at 490 nm using a universal microplate reader (Elx800, Bio-tek Instruments). IC50 values (the concentration that produced a 50 % reduction in absorbance) were analyzed and recorded. MTT assays were then performed for the proliferation of tumor cells treated with 5-aza-2-dC as described earlier. Briefly, ES2 and ESTR160 cells (1×10^4^ cells/well in a 6-well plate) with or without 10 μM 5-aza-2-dC (in growth media for 1, 3, 7, 10 or 14 days with 5 % CO_2_ at 37 °C). 5 mg/ml of MTT solution was then added to each well and incubated at 37 °C for 3 h. The medium was then aspirated and replaced with solubilization solution (DMSO). The plates were read on a Micro Elisa reader (Anthos 2001) at 570 nm.

### Cell cycle analysis of tumor cells treated with 5-aza-2-dC by flow cytometry analysis

To evaluate the influence of 5-aza-2-dC on the cell cycle of the tumor cells, the tumor cells were treated with or without 10 μM 5-aza-2-dC for 3 days, washed with PBS, and fixed with 70 % ethanol overnight at 4 °C. The cells were then stained with propidium iodide (50 μg/ml) and RNase A (100 μg/ml; Roth, Karlsruhe, Germany) for 20 min at 4 °C. The percentages of cells in the G_0_/G_1_ phase, S phase, and G_2_/M phase were determined using a FACScan flow cytometer and analyzed by Cell Quest software (Becton Dickinson, San Jose, CA).

### Caspase-3/7 activity of the tumor cells treated with paclitaxel and/or 5-aza-2-dC

Caspase-3/7 activity of the tumor cells was determined quantitatively using a Caspase-Glo 3/7 assay kit (Promega) according to the manufacturer’s instructions. Briefly, the tumor cell lines were seeded and treated with paclitaxel or 5-aza-2-dC. After 24 h, the cells were lysed and luminogenic substrates specific for the caspase species were added. Light emission was measured in a luminometer (Berthold Technologies, Wildbad, Germany).

### Genomic DNA and RNA extraction

Genomic DNA and RNA was extracted from the tumor cell lines using a QIAamp tissue kit (Qiagen, Valencia, CA) following the instructions of the manufacturer.

### Sodium bisulfite treatment, sequencing, and methylation-specific polymerase chain reaction analysis

Genomic DNA of the tumor cells was isolated using a Genomic DNA kit (Geneaid Biotech, Bade City, Taiwan), converted with sodium bisulfite using a CpGenome DNA modification kit (Millipore, MA, USA), purified, and then amplified by a PCR with DNA polymerase (ThermoHotStart 2× Gold PCR Master mix; Applied Biosystems) and HIN-1-specific primers. The primer sequences for methylated HIN-1 were 5’-GAAGTTTCGTGGTTTTGTTCG-3’ (forward) and 5’-AAAACCTAAAATCCACGATCGAC-3’ (reverse), and the primer sets for unmethylated HIN-1 were 5’-TAAGAAGTTTTGTGGTTTTGTTTGG-3’ (forward) and 5’-AAAAAACCTAAAATCCACAATCAAC-3’ (reverse). Bisulfite-modified Sss I (New England Biolabs, MA)-treated normal lymphocyte DNA served as the methylated control, and bisulfite-treated normal lymphocyte DNA as the unmethylated control. PCR products were analyzed on 3 % agarose gels. A methylation specific-PCR in a final volume of 20 μl was performed under the following conditions: 95 °C for 10 min, followed by 40 cycles at 95 °C for 30 s, 62 °C for 30 s, and 72 °C for 40 s, with a final extension at 72 °C for 10 min and holding at 4 °C. The PCR products were purified and then directly sequenced using an Applied Biosystems ABI automated DNA sequencer.

### Quantitative real-time RT-PCR (QRT RT-PCR)

QRT RT-PCR was used to measure the MDR1, NANOG, HIF-1α, HIF-2α, Snai2, TWIST1, and ABCG2 mRNA of the tumor cell lines. GAPDH was used as the internal control. The QRT RT-PCR was performed in an ABI Prism 7300 Sequence Detection System (Applied Biosystems) with a Taqman Gene Expression Assay (Hs00369360_g1) under the following conditions: 2 min at 50 °C, 10 min at 95 °C, and a two-step cycle at 95 °C for 15 s and 60 °C for 1 min for 40 cycles with an additional dissociation curve. The interpolated number (Ct) of cycles to reach a fixed threshold above background noise was used to quantify amplification.

### 5-aza-2-dC treatment and QRT RT-PCR of HIN-1 of the tumor cell lines

The tumor cell lines were treated with or without 5-aza-2-dC 10 μM, and renewed every 24 h. QRT RT-PCR was used to measure the mRNA of HIN-1 as described earlier.

### Immunohistochemistry

Formalin-fixed, paraffin-embedded specimens were sliced by a microtome at a thickness of 3–5 um and placed on coated slides. The tissue slides were then incubated with purified goat anti-human UGRP2 (S-15) polyclonal Ab (Santa Cruz Biotechnology) using a Thermo Scientific Autostainer 360 (Thermo Fisher Scientific Inc., CA). The immuno-reactive HIN-1 was scored semi-quantitatively, and the expression was scored according to the intensity as 0 or 1, 2 or 3 indicating no or low, intermediate or strong immuno-reactivity, respectively. Tissues containing more than 10 % neoplastic cells with a score of 2–3 intensity were considered to be positive. The percentages of each score in the neoplastic tissues were also recorded. If less than 10 % of the neoplastic cells expressed HIN-1 the expression was defined as being weak, and if more than 10 % of the neoplastic cells expressed HIN-1 the expression was defined as being strong. A pathologist not involved in the present study evaluated the immunostaining under blinded conditions.

### Western blot analysis

Tumor cell lines were first treated with paclitaxel or 5-aza-2-dC for 72 h. The cells were then collected and lysed in PBS containing 1 % Triton X-100 using an ultrasonic cell disruptor. The lysates were separated by SDS-PAGE (12.5 %) and transferred to a PVDF membrane. The membrane was blocked in blocking buffer (TBS containing 0.2 % Tween 20 and 1 % I-block (NEN)) and incubated with the polyclonal antibodies separately for 1 h. A purified rabbit anti-human GAPDH polyclonal Ab (Santa Cruz Biotechnology, Inc.) was also used at the same time to normalize the signals generated from anti-HIN-1, AKT, AKT p-Akt (Ser473), pAKT (Thr308), mTOR, and pMTOR (Cell Signaling). After washing, alkaline phosphatase-conjugated anti-rabbit Ab (Vector Laboratories) was applied. The membrane was washed and the bound Abs was visualized by developing with NBT/BCIP as chromogens.

### *In vivo* animal experiments

NOD/SCID (NOD.CB17 Prkdc ^scid^/Jnarl) mice were obtained from the National Animal Center (Taipei, Taiwan) and maintained in accordance with institutional policies. All of the experiments were approved by the Institutional Animal Care and Use Committee of Cathay General Hospital. Five to 7-week-old NOD/SCID mice (*n* = 4) were inoculated subcutaneously into the bilateral flank with 1 × 10^7^ of tumor cells treated with or without 10 μM 5-aza-2-dC for 3 days before inoculation. Tumor growth was measured using calipers, and volumes were calculated based on the modified ellipsoid formula (L × W × W/2) at the indicated time points. All of the experiments were carried out in duplicate.

### Statistical analysis

The median inhibitory concentrations (IC50) of paclitaxel were calculated using Sigma Plot 8.0 software (SPSS, Inc., Chicago, IL). All numerical data were expressed as the mean ± SD. Significance of the difference between two groups was determined with the Mann–Whitney *U* test. A *p* value less than 0.05 was considered to be statistically significant.

## Results

### Characteristics of paclitaxel-sensitive and paclitaxel-resistant cell lines in IC_50_, concentration, cell proliferation and distribution of cell cycle

**Fig. 1:**
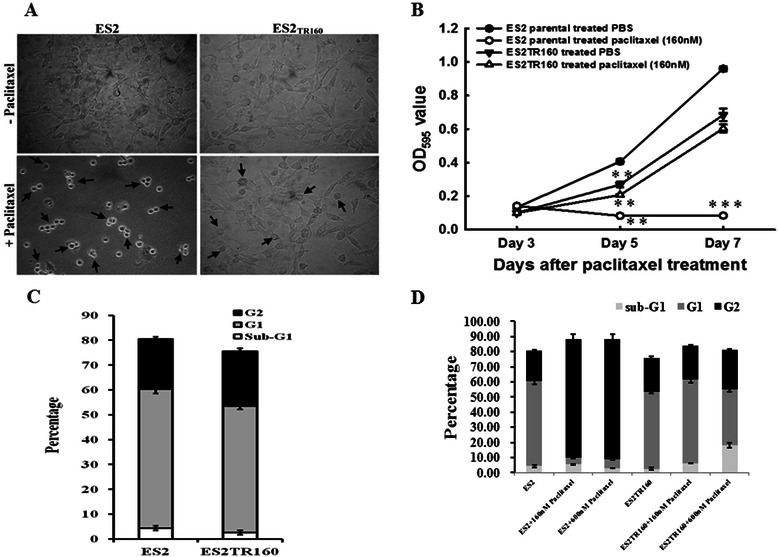
**a** Morphologic changes of ES2 and ES2TR160 cells before and after paclitaxel treatment. *Note:* There were more floating ES2 cells than floating ES2TR160 cells. **b** Cell growth curves of ES2 and ES2TR160 cells treated with or without 160 nM paclitaxel by MTT assays. **c** The percentages of sub-G1, G1 and G2 phases among parental ES2 and ES2TR160 cells analyzed by flow cytometry. **d** The percentages of sub-G1, G1 and G2 phases among parental ES2 and ES2TR160 cells treated with different concentrations of paclitaxel analyzed by flow cytometry

The IC50 concentrations of parental ES2 cells, TOV21G, and their paclitaxel-resistant clones ES2TR160 and TOV21GTR200 cells are shown in supplement Table 1. The relative resistant indices of ES2TR160 vs. ES2 and TOV21GTR200 vs. TVO21G were 9.36 and 228.3, respectively. The cell morphologies of the cell lines treated with paclitaxel are shown in Fig. 1a. Damaged morphology was noted in the ES2 cells but not in the ES2TR160 cells, including a decline in cell number, and rounded cells undergoing hydropic and vacuolated changes (Fig. 1a). Cell proliferation assays of ES2 and ES2TR160 cells treated with 160 nM of paclitaxel showed that the cell proliferative activity of the ES2 cells was significantly inhibited by paclitaxel compared with the ES2TR160 cells (Fig. 1b).

**Table 1:** Clinico-pathological characteristics and HIN-1 expression of 42 OCCC patients

Parameter	Low HIN-1 expression	High HIN-1 expression	*p* value
Patient numbers	18	24	
Age	49 (32–66)	55 (32–66)	0.393^a^
[years, median (range)]			
Disease stage			
Early (I + II)	12	4	0.067^b^
Advanced (IIII + IV)	14	12	
Tumor size (cm)	12.8 (6–21)	12.5 (3–23)	0.662^a^

*OCCC* ovarian clear cell carcinoma

^a^one-way ANOVA

^b^Chi-square test

The percentages of sub-G1, G1 and G2 phases among the parental ES2 and ES2TR160 cells treated with different concentrations of paclitaxel were further analyzed. There was no significant difference in the frequency of G1 (56.0 ± 1.8 % vs. 51.0 ± 1.4 %) or G2 (20.1 ± 0.9 % vs. 22.0 ± 1.3 %) phase in between the ES2 and ES2TR160 cells before treatment with paclitaxel (Fig. 1c), and the results were similar between TOV21GTR200 and TOV21G cells (data not shown). The percentage of the G2 phase in the ES2 cells treated with 160 nM paclitaxel was significantly higher than that in the ES2 cells without paclitaxel treatment (78.40 ± 3.35 % vs. 20.10 ± 0.88 %, *p* = 0.0001, one-way ANOVA) (Fig. 1d). In contrast, the percentage of the G2 phase in the ES2TR160 cells treated with 160 nM paclitaxel was not significantly different compared with that in the ES2TR160 cells without paclitaxel treatment (22.75 ± 0.44 % vs. 22.02 ± 1.27 %, *p* = 0.41, one-way ANOVA) (Fig. 1d). These results indicated that G2-M phase arrest was absent in the paclitaxel-resistant OCCC cells after being treated with a cytotoxic drug.

### Caspase activity in chemo-sensitive cells was higher than in chemo-resistant cells when treated with paclitaxel

**Fig. 2:**
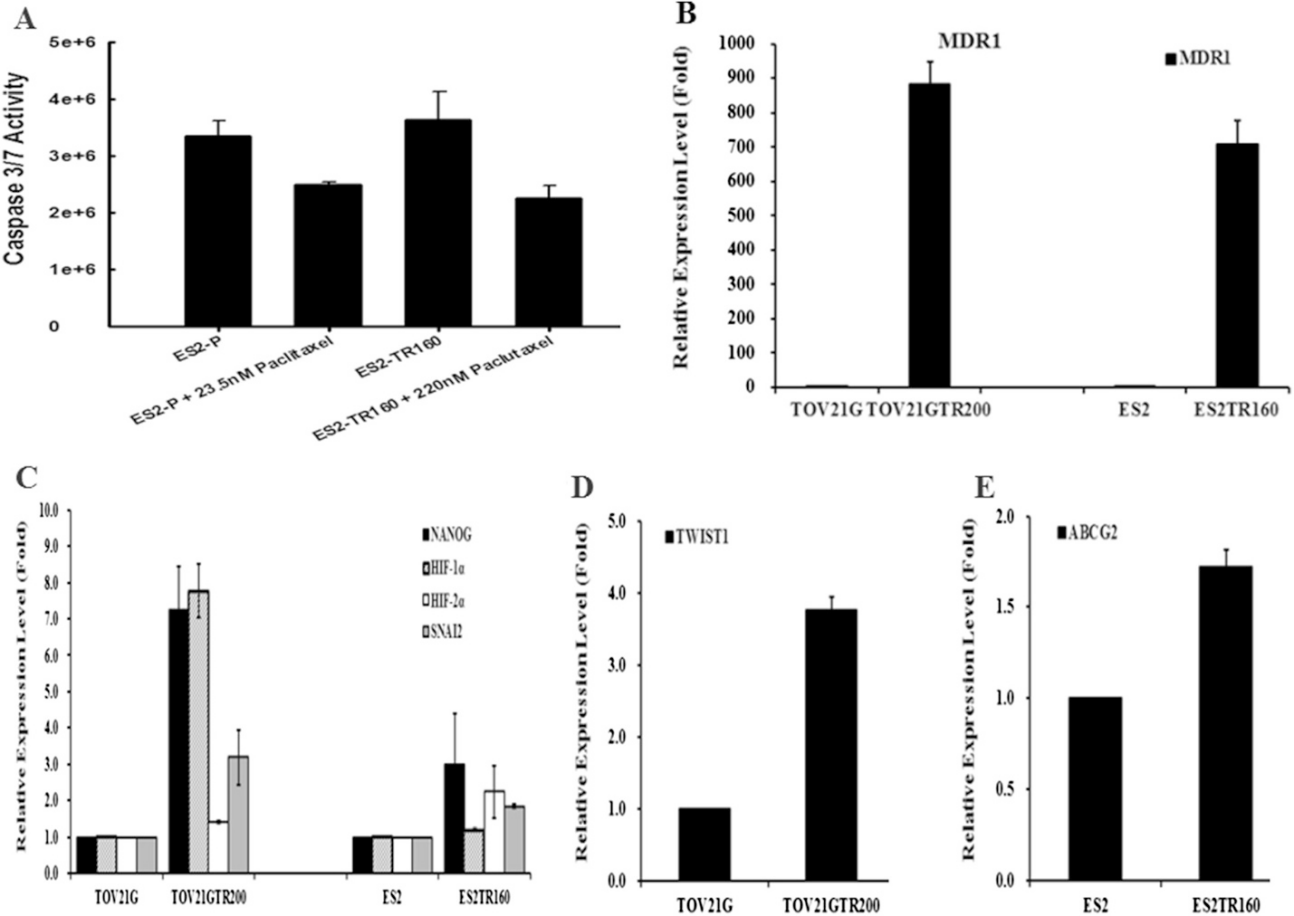
a Caspase-3/7 activity in both ES2 and ESTR160 cells with or without paclitaxel treatment. The expression levels of (**b**) MDR1, (**c**) NANOG, HIF-1α, HIF-2α, and Snai2, (**d**) TWIST1, and (**e**) ABCG2 in ES2 and TOV21G parental cells and their derived paclitaxel-resistant ES2TR160 and TOV21GTR200 cells by QRT-PCR

The caspase activity in the tumor cells treated with paclitaxel was then evaluated. As shown in Fig. 2a, the caspase-3/7 activity in the ES2 tumor cells was significantly higher than in the ESTR160 tumor cells when treated with paclitaxel (*p* < 0.001, one-way ANOVA).

### Drug resistance-related genes in paclitaxel-resistant tumor cells were more highly expressed than in paclitaxel-sensitive tumor cells

The expression levels of drug resistance-related genes were further evaluated by QRT RT-PCR. The expression levels of MDR1 (Fig. 2b), NANOG (Fig. 2c), HIF-1α (Fig. 2c), HIF-2α (Fig. 2c), Snai2 (Fig. 2c), TWIST1 (Fig. 2d), and ABCG2 (Fig. 2e) were significantly higher in the paclitaxel-resistant cell lines ES2TR160 and TOV21GTR200 than in the paclitaxel-sensitive cell lines ES2 and TOV21G. These results indicated that genes related to drug transport, cancer stem cell characteristics, hypoxic tumor microenvironment, and epithelial-mesenchymal transition were highly expressed in the paclitaxel-resistant tumor cells.

### HIN-1 methylation of paclitaxel-resistant tumor cells could be reversed by a demethylating agent

**Fig. 3:**
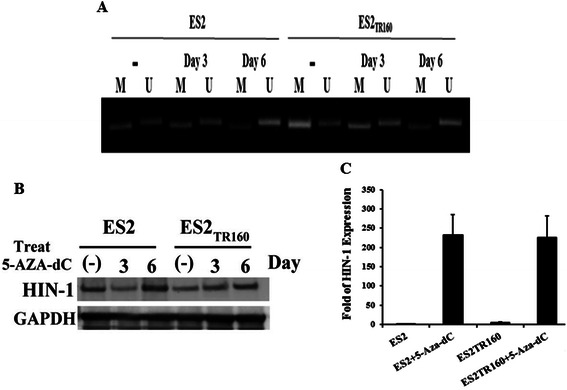
**a** Changes of methylation status of the HIN-1 gene detected by methylation-specific PCR in parental ES2 cells and the derived paclitaxel-resistant ES2TR160 cells before and after 5-aza-2-dC treatment for 3 or 6 days. M represents methylation and U represents unmethylation. **b** Representative figure of HIN-1 mRNA expression in ES2 and ES2TR160 cells treated with or without 5-aza-2-dC for 3 or 6 days. **c** Bar figure of the folds of HIN-1 mRNA expression in the ES2 and ES2TR160 cells treated with or without 5-aza-2-dC for 6 days

Changes in the methylation status of the HIN-1 gene in paclitaxel-sensitive and resistant tumor cell lines were evaluated by methylation-specific PCR. As shown in Fig. 3a, the ES2TR160 cells showed higher methylation of HIN-1 compared with the ES2 cells. We then tested whether a demethylating agent could reverse the methylation of the HIN-1 gene and then reactivate the expression of HIN-1. The methylation of HIN-1 in the ES2TR160 cells was reduced with 5-aza-2-dC treatment (Fig. 3a). In addition, the expression levels of HIN-1 in the 5-aza-2-deoxycytidine-treated groups were significantly increased compared with those in the PBS-treated groups in both the ES2 and ES2TR160 cell lines (Fig. 3b and c). These results indicated that the expression of HIN-1 in paclitaxel-resistant OCCC cells was lower than that in paclitaxel-sensitive OCCC cells due to the methylation of HIN-1. In addition, a demethylating agent could reverse the methylation of HIN-1 and restore its expression.

### Paclitaxel-resistant OCCC tissues expressed lower levels of HIN-1 than paclitaxel-sensitive OCCC tissues

**Fig. 4:**
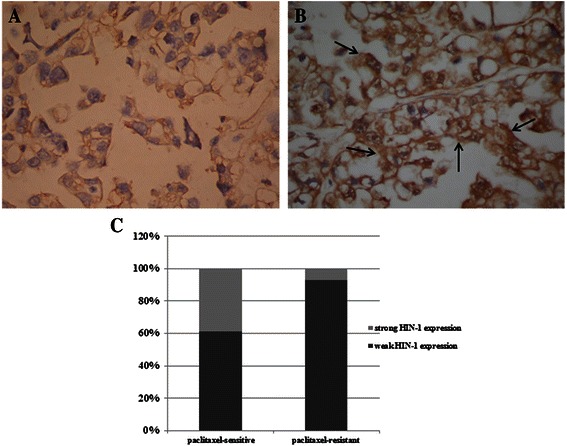
Representative immunohistochemical staining of HIN-1 in OCCC cancerous tissues. **a** Weak expression of HIN-1. **b** High expression of HIN-1. Note: The high HIN-1 expression was noted in the cytoplasm of the neoplastic cells (arrows). **c** Bar figures of the percentage of weak and strong HIN-1 protein expression in paclitaxel-resistant and paclitaxel-sensitive OCCC tissues

The representative photographs of HIN-1 immuno-reactivity in OCCC tumor tissues by immunohistochemical staining are shown in Fig. 4 (Fig. 4a: high expression of HIN-1, Fig. 4b: low expression of HIN-1). Fourteen (33.3 %) of 42 patients were paclitaxel-resistant and 28 (66.7 %) were paclitaxel-sensitive. Among the 14 paclitaxel-resistant OCCC tissues, 13 (93.8 %) showed a weak HIN-1 protein expression. In contrast, among the 28 paclitaxel-sensitive OCCC tissues, only 17 (62.8 %) showed a weak HIN-1 protein expression. The paclitaxel-resistant OCCC tissues had a significantly higher percentage of weak HIN-1 protein expression than the paclitaxel-sensitive OCCC tissues (93.8 % vs. 62.8 %, *p* = 0.03, chi-square test) (Fig. 4c). These results indicated that the HIN-1 expression was strongly associated with the response to paclitaxel of the OCCC patients. We retrospectively reviewed and analyzed our 42 OCCC patients. Among the analyzed 26 advanced OCCC tissues, 14 (54 %) samples of advanced OCCCs showed scored as HIN-1 weak staining (0 or +1 immuno-reactivity), and 12 (46 %) were HIN-1 strong staining (+2 or +3 immuno-reactivity). In contrast, among the 16 early stage OCCCs, 12 (75 %) tumors were scored as 0 or +1 immuno-reactivity and 4 (25 %) were +2 or +3. The percentage of HIN-1 immuno-reactivity at 0 or +1 was significantly higher in advanced OCCCs than in early stage (54 % vs 25 %, p = 0.067). These results indicate that loss of HIN-1 expression has a trend towards advanced OCCC tumors. However, HIN-1 expression levels among tumors are not associated with tumor size (*p* = 0.662) (Table 1).

### HIN-1 reduced *in vivo* tumor growth

**Fig. 5:**
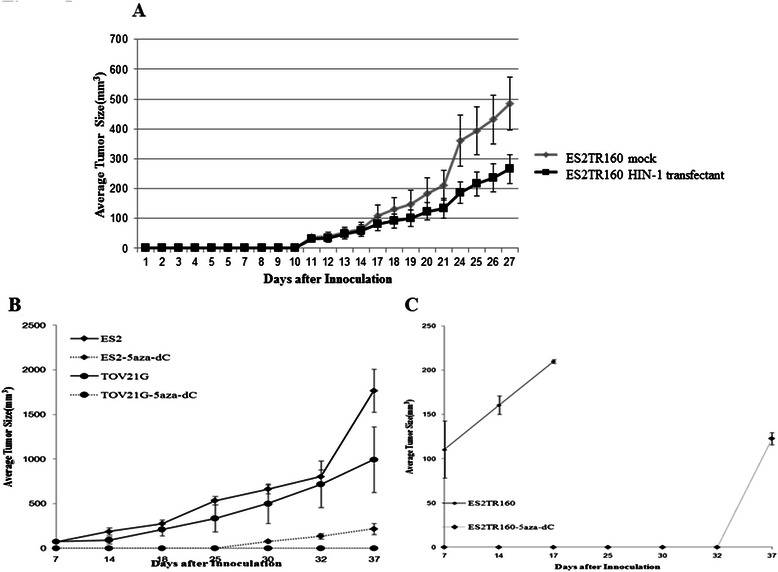
**a** The average tumor size in xenograft mice after subcutaneous inoculation of 1 × 10^6^ cells of ES2TR160 mock or ES2TR160 HIN-1 transfectants. **b** The average tumor sizes in xenograft mice after subcutaneous inoculation of 1 × 10^6^ of ES2 or TOV21G cells with or without 5-aza-2-dC treatment. **c** The average tumor sizes in xenograft mice with subcutaneous inoculation of 1 × 10^6^ of ES2TR160 cells with or without 5-aza-2-dC treatment

To further examine whether HIN-1 could inhibit the growth of paclitaxel-resistant OCCC tumor cells, in vivo subcutaneous xenograft experiments were performed. Mice receiving ES2TR160 cells expressing high concentrations of HIN-1 had a smaller tumor size compared with those challenged with ES2TR160 parental cells (Fig. 5a) (ES2TR160 cells with high expressions of HIN-1 vs. ES2TR160 mock cells; day 21, 133.76 vs. 211.74 mm^3^, *p* = 0.036; day 27, 266.55 vs. 484.92 mm^3^, *p* = 0.008, both by the Student’s *t* test). These results indicated that HIN-1 could inhibit the in vivo growth of paclitaxel-resistant OCCC tumor cells.

### 5-Aza-2-dC inhibited the *in vivo* tumor growth of paclitaxel-sensitive and resistant OCCC cell lines

The *in vivo* growth inhibitory effect of 5-aza-2-dC on OCCC tumor cells was further evaluated. As shown in Fig. 5b, the mice receiving ES2 or TOV21G parental tumor cells treated with 5-aza-2-dC had smaller tumor sizes compared with those treated with PBS. The mean tumor size of the ES2 tumor cells treated with 5-aza-2-dC was smaller than that of ES2 tumor cells treated with PBS (day 37, 217.8 vs. 1764.1 mm^3^, *p* < 0.01, Student’s *t* test). In addition, the mice challenged with ES2TR tumor cells with PBS died on day 18. However, none of the mice challenged with ES2TR tumor cells treated with 5-aza-2-dC had died 35 days after tumor challenge (Fig. 5c). These results suggest that 5-aza-2-dC effectively inhibited the growth of both paclitaxel-sensitive and resistant OCCC tumor cells.

### 5-Aza-2-dC inhibited the proliferative activities of both paclitaxel-sensitive and resistant OCCC cell lines *in vitro*

**Fig. 6:**
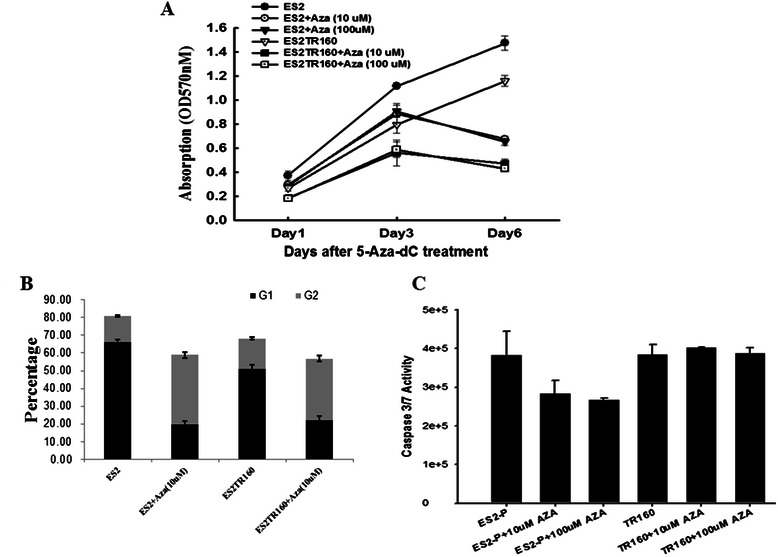
**a** The effect of 10 μM or 100 μM of 5-aza-2-dC on *in vitro* cell growth of ES2 and ES2TR160 cells by MTT assay. **b** The percentages of G1 and G2 phases in both ES2 and ES2TR160 cells with 5-aza-2-dC treatment for 3 days. **c** The caspase-3/7 activity in both ES2 and ES2TR160 cells with or without 5-aza-2-dC treatment

The effects of 5-aza-2-dC on the growth of paclitaxel-sensitive and paclitaxel-resistant OCCC cells were examined by MTT assays. The results showed that 5-aza-2-dC inhibited cell growth by 70 % in both ES2 and ES2TR160 tumor cells (Fig. 6a). In addition, 5-aza-2-dC significantly reduced the percentages of the G1 phase (66.4 % ± 1.1 to 19.9 % ± 1.8 % in the ES2 cells, *p* < 0.001; 50.8 % ± 2.7 to 22.0 % ± 2.4 % in the ES2TR160 cells, *p* < 0.001, Student’s *t* test), but significantly increased the percentages of the G2 phase (14.4 % ± 0.5 to 39.0 % ± 1.7 % in the ES2 cells, *p* < 0.001; 17.4 % ± 0.6 to 34.8 % ± 1.8 % in the ES2TR160 cells, *p* < 0.01, Student’s *t* test) after 3 days of treatment (Fig. 6b). Furthermore, 5-aza-2-dC also reduced the caspase-3/7 activity in ES2 cells but not in ES2TR160 cells (Fig. 6c).

### HIN-1-AKT-mTOR signaling pathway was involved in the paclitaxel-treated OCCC tumor cells

**Fig. 7:**
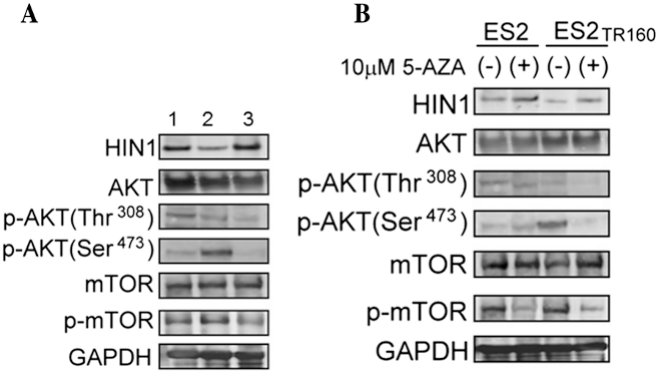
**a** Changes in HIN-1, p-AKT (Thr308), p-AKT (Ser473), and p-mTOR protein expressions in ES2 mock, ES2TR160 mock, and ES2TR160HIN-1 transfectants by Western blotting. 1: ES2 mock, 2: ES2TR160 mock, 3: ES2TR160HIN-1 transfectant. **b** HIN-1, p-AKT (Thr308), p-AKT (Ser473), and p-mTOR protein expressions in ES2 and ES2TR160 cells treated with or without 5-aza-2-dC by Western blotting

The molecules involved in the signaling pathways associated with paclitaxel-related drug resistance were further evaluated by immunoblotting analysis. The expressions of phospho-AKT473 and phospho-mTOR were significantly increased in the ES2TR160 cells in parallel to the decrease in HIN-1 expression compared to the ES2 parental cells (Fig. 7a). However, the expressions of phospho-AKT at Ser473 and Thr308 and phospho-mTOR were decreased in the ES-2TR160 tumor cells with high expressions of HIN-1 (Fig. 7a). In addition, 5-aza-2-dC also decreased the expressions of phospho-AKT at Ser473 and Thr308 and phospho-mTOR. Whereas, an increased HIN-1 expression was observed in the 5-aza-2-dC-treated paclitaxel-resistant ES2TR160 cells (Fig. 7b). These results demonstrated that 5-aza-2-dC may increase the expression of HIN-1 by decreasing the AKT-mTOR expression in paclitaxel-resistant OCCC tumor cells.

## Discussion

Aberrations in DNA methylation are involved in tumor progression and the acquisition of drug resistance. To investigate whether paclitaxel selects preferentially for DNA methylation in chemo-resistant OCCC cell lines, we used MS-MLPA to detect 40 tumor suppressing genes (TSGs) in ES2 and TOV21G parental and resistant cells. We found changes in methylation of the HIN-1 gene in ES2TR160 and TV21GTR200 paclitaxel-resistant cells, and this may be involved in the mechanism of paclitaxel resistance (data not shown). Significantly more paclitaxel-resistant OCCC cells had a low expression of the HIN-1 protein compared to the paclitaxel-sensitive OCCC cells (93.8 % vs. 62.8 %, *p* = 0.03), suggesting that down-regulation of the expression of HIN-1 is strongly correlated with paclitaxel-resistant OCCC tumors. Over-expression of the HIN-1 gene effectively decreased the tumor growth of paclitaxel-resistant ES2TR160 tumor cells, which is consistent with promoter methylation of HIN-1 and the poor outcomes of patients with OCCC [16]. 5-Aza-2-dC inhibited tumor growth by demethylating aberrantly methylated TSGs and maintaining function, presumably through restoration of HIN-1 expression with a decrease in AKT-mTOR expression. Furthermore, 5-aza-2-dC inhibited growth of ES2 and ES2TR160 cells mainly by inhibiting the G2M phase, but without increasing apoptosis and autophagy (Fig. 6). These results support the concept that 5-aza-2-dC can inhibit tumor growth of OCCC partly through affecting the HIN-1-related AKT-mTOR signaling pathway, and this may be a promising therapy for the management of primary or recurrent OCCC. Clinical trials are warranted to test this hypothesis.

OCCC is a chemo-resistant tumor. Experimental evidence has suggested different mechanisms by which tumor cells can develop resistance to taxanes, including excluding taxane from cells by ATP-binding cassette transporters, the expression of certain tubulin isoforms and microtubule associated proteins, tubulin gene mutations, alterations in survival or mitotic check point signaling, and methylation-associated Has-miR-9 deregulation [17, 18]. ES2TR160 and TOV21GTR200 cancer cell lines did not acquire paclitaxel resistance via somatic mutations in tubulin genes, as those nicely reviewed in previous report [18]. In the present study, however, G2-M phase arrest was absent in the paclitaxel-resistant OCCC cells even though these cells were under paclitaxel treatment. Our results support that the mechanism of paclitaxel resistance in OCCC may involve the drug transporter gene, cancer stem cell characteristics, hypoxic tumor microenvironment, and epithelial-mesenchymal transition.

Methylation of HIN-1 is involved in the chemo-resistance of OCCC. Importantly, the reversal of HIN-1 epigenetic silencing by demethylation or over-expression of the HIN-1 gene was demonstrated to resensitize tumor cells to paclitaxel treatment in vitro and in vivo. We also observed that CpG sites at probe 12956 of the HIN-1 gene were hypomethylated in ES2 and TOV21G cells, whereas they became hypermethylated following step-wise exposure to paclitaxel in ES2TR160 and TOV21GTR200 cells as confirmed by methylation-specific PCR, suggesting that hypermethylation occurs in acquired paclitaxel chemo-resistance (data not shown).

We previously showed that the ectopic expression of the HIN-1 gene increases paclitaxel sensitivity, partly through the Akt pathway [16]. The SCGB3A1 gene, also called HIN-1 (high in normal-1), encodes a small secreted protein, secretoglobin 3A1 which is a member of the secretoglobin family [19]. Recent reports have shown that HIN-1 expression is down-regulated in the majority of lung, breast, prostate, pancreatic, colorectal, testicular and nasopharyngeal cancers, and that this down-regulation is associated with hypermethylation of the HIN-1 promoter [20–24]. Thus, silencing of HIN-1 expression by methylation is an early and frequent event in multiple human types of cancer, and is functionally relevant to tumorigenesis [22]. These findings together with *in vitro* data on growth inhibition and AKT activation in breast cancer suggest that HIN-1 may be a candidate tumor suppressor gene [24].

Clinical studies have shown that a low dose of decitabine can alter the DNA methylation of genes and cancer pathways, thereby restoring sensitivity to carboplatin in heavily pretreated ovarian cancer patients who progressed or recurred within 6 months after platinum-based chemotherapy, resulting in a high response rate and prolonged progression free survival [12]. The selective epigenetic disruption of distinct biological pathways has been observed during the development of platinum resistance in patients with ovarian cancer. Hypermethylation-mediated repression of cell adhesion and tight junction pathways, and hypomethylation-mediated activation of the cell growth-promoting pathways PI3K/Akt and TGF-beta, and cell cycle progression may contribute to the onset of chemo-resistance in ovarian cancer cells [15].

The PI3K/Akt pathway has been shown to contribute to cisplatin resistance by promoting cell proliferation and increasing drug metabolism and resistance to apoptosis [25, 26]. Paclitaxel activates AKT and mTORC1 signaling which act as resistant factors and protect cancer cells from death/apoptosis [27, 28]. The mammalian target of rapamycin (mTOR) has been identified to be a downstream target of the PI3K/Akt pathway, and it has emerged as a critical effector in cell signaling pathways commonly deregulated in human cancers. mTOR has been reported to be phosphorylated and activated in endometriosis and OCCC specimens [29]. This leads to phosphorylation of downstream targets, p70S6K and 4E-BP1, and the subsequent enhanced translation of mRNA that is critical for cell cycle progression and proliferation. Recently, a therapeutic strategy targeting the mTOR-HIF-1α-VEGF pathway in OCCC has been proposed based on the finding that p-mTOR expression is more prominent in OCCC than ovarian serous carcinoma [30]. After treatment with an analogue of rapamycin (everolimus), the expressions of p-mTOR, HIF-1α and VEGF were shown to be sharply decreased [30].

In this study, 5-aza-2-dC not only decreased phospho-AKT at Thr308 and Ser473 and phospho-mTOR, but also restored HIN-1 expression in paclitaxel-resistant cells *in vitro*. In addition, treatment with 10 μM 5-aza-2-dC also inhibited the growth of both ES2 and ES2TR160 tumor cells (Fig. 6a). Furthermore, 5-aza-2-dC treatment significantly reduced the percentage of the G1 phase in both ES2 and ES2TR160 cells after 3 days of treatment. These results support that 5-aza-2-dC can overcome paclitaxel drug resistance and inhibit tumor growth of paclitaxel-resistant OCCC partly through affecting the HIN-1-AKT-mTOR signaling pathway.

Taken together, a demethylating agent can restore the HIN-1 expression in paclitaxel-resistant OCCC cells through the HIN-1-AKT-mTOR signaling pathway and then inhibit in vivo tumor growth. Restoration of HIN-1 by a demethylating agent may be a potential strategy for the treatment of paclitaxel-resistant OCCC.

## Conclusion

This is the first study to show that a demethylating agent could inhibit tumor growth of OCCC *in vivo* and *in vitro*. Treatment with 5-aza-2-dC remarkably inhibited ES2-derived tumor growth by 90-100 % compared to the controls, in both ES2 (paclitaxel-sensitive) and ES2TR160 (paclitaxel-resistant) cells treated with 5-aza-2-dC for 3 days prior to subcutaneous inoculation in mice. We concluded that 5-aza-2-dC has a remarkable anti-tumor effect as a single agent in OCCC.

## Abbreviations

OCCC: Ovarian clear cell carcinoma
5-aza-2-dC: 5-aza-2-deoxycytidine
